# Does problem-based learning education improve knowledge, attitude, and perception toward patient safety among nursing students? A randomized controlled trial

**DOI:** 10.1186/s12912-021-00588-1

**Published:** 2021-04-29

**Authors:** Hossein Jamshidi, Masumeh Hemmati Maslakpak, Naser Parizad

**Affiliations:** 1 School of Nursing & Midwifery, Urmia University of Medical Sciences, Urmia, Iran; 2 Maternal and Childhood Obesity Research Center, Nursing and Midwifery School, Urmia University of Medical Sciences, Urmia, Iran; 3 Patient Safety Research Center, Clinical Research Institute, Nursing and Midwifery School, Urmia University of Medical Sciences, Urmia, Iran

**Keywords:** Education, Problem-based learning, Patient safety, Knowledge, Attitude, Perception, Nurse, Student

## Abstract

**Background:**

Patient safety is a top priority for any health care system. Most universities are looking for teaching methods through which they would be able to enhance students’ clinical decision-making capabilities and their self-centered learning to ensure safe and quality nursing care. Therefore, this study aimed to determine the effect of patient safety education through problem-based learning (PBL) on nursing students’ knowledge, attitude, and perceptions toward patient safety.

**Methods:**

This randomized, controlled trial was conducted from September 2019 to January 2020. A total of 78 fourth-year nursing students participated in this study. The participants were randomly assigned to either the intervention group or the control group. In the intervention group, the educational materials were presented to the students using the PBL method during eight sessions of 45–60 min. In each control group, nursing students received eight education sessions through lectures and discussing the same educational content. Data were gathered 1 month after the intervention using demographic information and knowledge, attitudes, and perception questionnaires. Data were analyzed in SPSS ver. 22.0 using descriptive (mean and standard deviation) and inferential (chi-square test, independent t-test, paired t-test, and analysis of covariance (ANCOVA)) statistics.

**Results:**

The results indicated that the difference in the mean scores of knowledge, attitudes, and perceptions of the nursing students about patient safety was statistically significant between the two groups after the PBL education (*p* = 0.001). The mean scores of students’ knowledge, attitude, and perceptions of patient safety increased significantly in the intervention group.

**Conclusions:**

Implementing patient safety education through PBL positively affects knowledge, attitudes, and perceptions of patient safety among nursing students. Thus, the research team recommended the PBL method to be used by nursing professors to improve nursing students’ clinical skills and cognitive abilities to ensure safe patient care.

**Trial registration:**

IRCT20190925044881N1; October 17, 2019.

**Supplementary Information:**

The online version contains supplementary material available at 10.1186/s12912-021-00588-1.

## Background

Patient safety is a priority issue for all health care systems worldwide [1, 2]. Providing safe and error-free care is the ultimate goal of all healthcare systems [3]. Nurses are leading healthcare team members [4], and they have a fundamental responsibility to ensure patient safety [5]. It is estimated that there are 421 million hospital admissions worldwide every year. Meanwhile, approximately 7.42 million cases of adverse events occur during these hospitalizations, making patient harm is the 14th leading cause of global deaths [6]. Furthermore, one in every ten patients is harmed while receiving hospital care as the world health organization (WHO) considers patient safety as an endemic and epidemic concern [7]. Annually, more than 400,000 premature deaths occur due to preventable adverse events, and the incidence of serious harm is 10 to 20 times higher than the mortality rate [8]. In clinical settings, nursing students sometimes participate directly in unsafe care, errors, adverse events, and poor patient care. For that reason, like other healthcare team members, they should use their knowledge, attitude, and perception of patient safety while caring for the patient [9]. Lack of patient safety knowledge is one of the nursing students’ educational problems that lead to unsafe practice [10]. Mansour and Francis (2013) stated that graduate nurses should have sufficient knowledge to identify potential safety risks, and they should have the confidence to protect patients against preventable harm or adverse events [11, 12].

On the other hand, an unsafe attitude is a precursor to adverse events because it shapes and influences the behavior, so any change in attitude has a significant effect on people’s safety behavior [13]. Nowadays, it is widely accepted that optimal patient safety development is not possible without a safe attitude in health care facilities [14]. Therefore, nurses’ attitude toward patient safety is very important to promote a safe environment for patients [15]. Nurses’ perceptions are the foundation of any behavior and lead to actions that affect patient safety and are vital for all hospitals and healthcare providers [16].

Hence, evaluating nursing and medical students’ knowledge, attitude, and perceptions toward patient safety is necessary because they are future healthcare professionals [17]. Most universities around the world are looking for teaching methods through which they would be able to enhance students’ clinical decision-making capabilities and self-centered learning [18]. In recent decades, the use of new and active student-centered learning methods has been trending strongly with educational systems [19]. The PBL is an innovative educational method that focuses on one problem, either assigned by the students or by the teacher [20], and it has been adopted in medical sciences such as nursing, midwifery, dentistry, and medicine in many universities around the world [21]. The PBL is a student-centered pedagogy in which students and professors are responsible partners in the learning-teaching process, and teaching is a way to facilitate learning [22]. The purpose of this method in medical education is to acquire basic clinical knowledge, make progress in personal learning skills, and evolve in dealing effectively with challenges at the patient’s bedside, and ultimately improve dynamism and motivation for learning [23].

As members of the healthcare team, nurses play a vital role in improving patient safety, originating from their attitudes, knowledge, and skill in patient safety [15, 24]. Also, the WHO emphasizes teaching patient safety to medical and nursing students, and the ministries of health focus on patient safety programs [25]. Given the widespread adoption of PBL in medical and nursing schools worldwide and many nursing education, experts believe that PBL can bridge the gap between theory and practice [26, 27]. Thus, this study aimed to determine the effect of patient safety education through PBL on nursing students’ knowledge, attitude, and perceptions toward patient safety.

## Methods

### Research design and setting

This randomized, controlled trial was conducted in the Urmia School of Nursing and Midwifery from September 2019 to January 2020. This study was permitted by the Review Board of Urmia University of Medical Sciences (IR.UMSU.REC.1398.219) and obtained a registration code from the Iranian registry of clinical trials (IRCT20190925044881N1).

### Study participants

In this study, all fourth-year nursing students who met the inclusion criteria constituted the study population. The inclusion criteria consisted of the following: (i) willing to participate in the study, (ii) being a fourth-year nursing student, and (iii) having no involvement in the same educational programs. The exclusion criteria included the following: (i) unwilling to stay in the research, and (ii) having more than two absences from the educational sessions. Based on the previous similar study (the mean and standard deviation of the problem-based learning score was 6 ± 2.14 and 7.76 ± 2.18 in the control and intervention groups, respectively) and considering the effect size (ES) = 0.814, α = 0.05, and power of 90%, the sample size was measured 32 for each group [28]. A total of 78 fourth-year nursing students were recruited into the study to consider a drop-out rate of 10%. Cohen (1992) suggested that an effect size of 0.80 is large enough to enable us to compare an experiment’s effect-size findings to a known benchmark [29].

$$ n=\frac{{\left({Z}_{1-\frac{\alpha }{2}}+{Z}_{1-\beta}\right)}^2\left({\delta}_1^2+{\delta}_2^2\right)}{{\left({\mu}_1-{\mu}_2\right)}^2} $$

$$ {\mathrm{n}}_1={\mathrm{n}}_2=\frac{{\left(1.96+1.28\right)}^2\left({2.18}^2+{2.14}^2\right)}{{\left(7.76-6\right)}^2}=31.63\cong 32 $$

### Randomization

The department manager had divided the participants into nine groups based on the internship curriculum. The second researcher randomly allocated the participants into five intervention (*n* = 43) and four control (*n* = 35) groups. The simple randomization was used to allocate nursing students to either control or intervention groups. The random allocation was as follows: the first researcher assigned a name to each of the nine groups and placed the groups’ names inside opaque envelopes. The first five groups picked from the envelope were considered the intervention groups. The remaining four groups were recognized as the control groups.

### Outcome measure

A two-part questionnaire was used to collect data: a demographic information questionnaire and a questionnaire on nursing students’ knowledge, attitude, and perceptions toward patient safety. It was adopted from Leung’s (2010) [30] and Madigoskay et al. (2006) [31] studies. This questionnaire comprises 26 questions, of which six questions assess students’ knowledge about patient safety (primary outcome), eight questions assess their attitude or tendency towards patient safety (secondary outcome), and 12 questions assess students’ perception of patient safety (secondary outcome). This questionnaire is scored on a 5-point Likert scale. In section 1 (attitude and perception items) of the questionnaire on patient safety, the 5-point Likert scale is scored as follows: 1 = strongly disagree, 2 = disagree, 3 = neutral, 4 = agree, 5 = strongly agree, and in section 2 (knowledge items), this scale is scored as follows: 1 = very poor, 2 = poor, 3 = fair, 4 = good, 5 = very good. In a study by Nabilou et al. (2015), this questionnaire was first translated into Persian, and then it was re-translated into English (forward and backward translation method) and reviewed by two faculty members who were skillful at the English language. Ultimately, the necessary adaptations were made to the questionnaire. The research team and four patient safety experts reviewed the questionnaire and confirmed its validity. The reliability of the questionnaire was also confirmed using the internal consistency method with Cronbach’s alpha of 0.723 [25].

### Study interventions

#### The PBL-based education

Implementing the educational process was such that in each session, the first researcher presented a written scenario to the students about knowledge, attitude, and perception of patient safety in the intervention group. Students had a week to review the scenario. A problem-based learning method was implemented to investigate each scenario in the intervention group. The PBL method’s steps were as follows: In the first step, the instructor asked the students to read the problem scenario and encouraged them to clarify vague concepts. In the second step, the problem was defined by the instructor. In the third step, the students had the brainstorming and group discussion about the problem. In the fourth step, students listed the facts, generated hypotheses based on the scenario content, and answered the questions based on the nursing process to achieve educational goals. In the fifth step, they reached a consensus on learning objectives within the group, and the instructor assured them in achieving complete, comprehensive, and appropriate goals. In the sixth step, they conducted independent and group study to gather information by using the library and the internet from resources introduced. In the seventh step, the instructor presented and analyzed the solutions based on the hypotheses, goals, and questions, conducted the interdisciplinary discussion, summarized and evaluated the proposed solutions (Table 1).

**Table 1:** Steps of the PBL-based intervention

*1st step*	Reading the problem scenario and encouraging students to clarify vague concepts
*2nd step*	Defining the problem by the instructor
*3rd step*	Brainstorming and group discussion about the problem
*4th step*	Listing facts and generating hypotheses based on the scenario content, and answering the questions based on the nursing process to achieve educational goals.
*5th step*	Reaching a consensus on learning objectives within the group and the instructor assurance of achieving complete, comprehensive and appropriate goals
*6th step*	Conducting independent and group study to gather information using the library and the internet from resources introduced
*7th step*	Presenting and analyzing the solutions based on the hypotheses, goals and questions, conducting interdisciplinary discussion, summarizing and evaluating the presented solutions by the instructor

In each of the five intervention groups, eight education sessions of 45–60 min were conducted. Then, a total of 40 sessions was carried out using the PBL in this study. The instructor reviewed the scenario delivered to the students last week at the beginning of each session. At the end of the session, the instructor presented the following week’s scenario to the students.

#### Routine education

In the control groups, the researcher performed routine education to teach the same educational content regarding patient safety. The hospital’s routine method was to lecture and discuss educational content. The students had eight routine sessions in each control group. A total of 32 sessions took place in the control groups (See supplementary file).

### Data collection procedure

The second researcher held the introductory session at Urmia School of Nursing and Midwifery. He introduced himself to the participants and presented the study process and objectives for them. Participants completed questionnaires after they signed a written informed consent form. The study intervention lasted for 4 months. Then, all the participants filled in the questionnaire on patient safety 1 month after the intervention. The second researcher held the PBL educational sessions for nursing students in the control group after the intervention finished.

### Data analysis

**Fig. 1:**
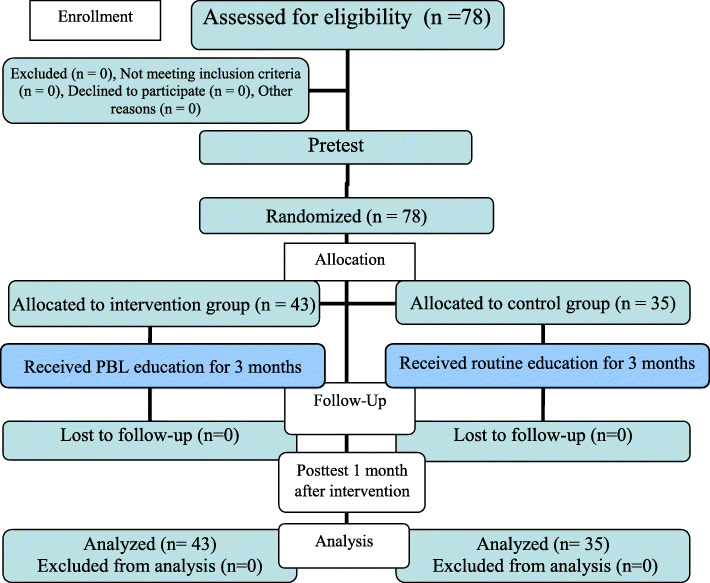
CONSORT flow diagram of the study

Collected data were entered into SPSS software version 22.0 (IBM Corp., Armonk, NY. USA) and analyzed using descriptive (mean and standard deviation) and inferential (chi-square test, independent t-test, paired t-test, and analysis of covariance (ANCOVA) statistics. The CONSORT flow diagram of the study is presented in Fig. 1. The CONSORT 2010 checklist was utilized to ensure quality reporting in the present study [32].

## Results

Seventy-eight nursing students entered the analysis, with no attrition in this study. The results indicated no statistically significant difference between the two groups in terms of age, gender, semester, marital status, residency, interest in the nursing major, clinical work experience, and grade point average (GPA), indicating that the two groups were homogeneous (Table 2).

**Table 2:** Demographics of the participants in the intervention and control groups

Variable		Control group	Intervention group	*Statistic test*
		Frequency (*percent*)	Frequency (*percent*)	
Gender	Male	12 (*33.3*)	24 (*66.7*)	*x*^2^*=3.59* ^*b*^*p = 0.058*
	Female	23 (*54.8*)	19 (*45.2*)	
Semester	7th	14 (*38.9*)	22 (*61.1*)	*x*^2^*=0.96* ^*b*^*p = 0.325*
	8th	21 (*50*)	21 (*50*)	
Marital status	Single	32 (*47.1*)	36 (*52.9*)	*x*^2^*=1.02* ^*b*^*p = 0.311*
	Married	3 (*30.0*)	7 (*70.0*)	
Residency	Dormitory residence	23(*45.1*)	28 (*54.9*)	*x*^2^*=0.003* ^*b*^*p = 0.956*
	Non-dormitory residence	12 (*44.4*)	15 (*55.6*)	
Interest in nursing major	Interested	22 (*45.8*)	26 (*54.2*)	*x*^2^*=0.47* ^*b*^*p = 0.849*
	Uninterested	13 (*43.3*)	17 (*56.7*)	
Work experience	Yes	3 (*60.0*)	2 (*40.0*)	*x*^2^*=0.494* ^*b*^*p = 0.482*
	No	32 (*43.8*)	41 (*56.2*)	
Mean age (year)		23.26 ± 1.40	22.95 ± 1.64	*t = −0.756* ^*a*^*p = 0.390*
Grade point average (GPA)		16.01 ± 0.77	16.08 ± 1.14	*t = 0.364* ^*a*^*p = 0.717*

^a^Independent t-test

^b^Chi-square test

The results of the paired t-test indicated that the mean score of patient safety knowledge in the control group did not differ significantly before and after the intervention (*p* = 0.279). However, the mean score of patient safety knowledge in the intervention group increased significantly after the intervention (*p* = 0.001) (primary outcome). Moreover, based on the paired t-test result, the mean score of students’ attitudes toward patient safety was not significantly different in the control group after the PBL education (*p* = 0.529). However, the difference was statistically significant in the intervention group after the PBL education as the mean score of students’ attitudes about patient safety increased significantly after the intervention (*p* = 0.016) (secondary outcome). The paired t-test also showed no significant difference in the mean score of the students’ perception of patient safety in the control group before and after PBL education (*p* = 0.122). Nevertheless, the mean score of students’ perception of patient safety increased significantly in the intervention group after PBL education (*p* = 0.037) (secondary outcome) (Table 3).

**Table 3:** Comparison of the mean scores of patient safety knowledge, Patient safety attitude, and the perception of Patient safety between and within the intervention and the control group before and after the intervention

Variables		Control group	Intervention group	Independent t-test
		SD ± Mean	SD ± Mean	
Patient safety knowledge	Before the intervention	10.28 ± 3.18	14.97 ± 3.70	*t = 0.591* *p = 0.001*
	After the intervention	11.05 ± 4.24	19.37 ± 3.31	*t = 9.71* *p = 0.001*
Paired t-test		*t = −1.09* *p = 0.279*	*t = −9.52* *p = 0.001*	
attitude toward Patient safety	Before the intervention	30.11 ± 4.36	31.74 ± 5.38	*t = 1.44* *p = 0.152*
	After the intervention	29.77 ± 3.99	34.62 ± 9.59	*t = 2.80* *p = 0.006*
Paired t-test		*t = 0.636* *p = 0.529*	*t = −2.50* *p = 0.016*	
The perception of Patient safety	Before the intervention	33.88 ± 5.56	35.60 ± 7.51	*t = 1.12* *p = 0.264*
	After the intervention	32.88 ± 5.09	38.95 ± 8.21	*t = 3.81* *p = 0.0001*
Paired t-test		*t = 1.58* *p = 0.122*	*t = −2.15* *p = 0.037*	

A significant difference was found in the mean score of patient safety knowledge between the control and the intervention group before and after the intervention (*p* = 0.001). No statistically significant difference was revealed in the mean score of students’ attitudes toward patient safety between the two groups before the intervention (*p* = 0.152). However, the difference was statistically significant between the two groups after the PBL education (*p* = 0.006). Consequently, the PBL positively affected students’ attitudes about patient safety in the intervention group. The independent t-test demonstrated that the difference in the mean score of the students’ perception of patient safety was not statistically significant between the two groups before the intervention (*p* = 0.264). Moreover, after the intervention, the mean score of students’ perception toward patient safety increased significantly in the intervention group compared to the control group (*p* = 0.001). Accordingly, the PBL had a positive effect on the mean score of students’ perceptions of patient safety in the intervention group.

Because there was a significant difference in the mean score of patient safety knowledge between the two groups before the intervention (Table 3), we used ANCOVA analysis to ensure that the significant difference in the mean score of patient safety knowledge after the intervention is due to the PBL educational approach, not the effect of the pre-interventional knowledge in the intervention group. After checking Levene’s test to confirm the homogeneity of variance between the two groups, we used ANCOVA analysis and confirmed the effect of PBL on the mean score of knowledge differences between the two groups after intervention (f = 40.90, *p* < 0.05) (Table 4).

**Table 4:** Results of ANCOVA test for mean scores of knowledge in control and intervention groups

	Total squares	Degree of freedom	Mean Square	*f*	*sig.*
*Modified model*	*1619.19*	*2*	*809.59*	*98.76*	*p = 0.001*
*Internal effect*	*360.68*	*1*	*360.68*	*29.34*	*p = 0.001*
*Pre-interventional knowledge*	*285.17*	*1*	*285.17*	*27.11*	*p = 0.001*
*Groups*	*430.17*	*1*	*430.17*	*40.90*	*p = 0.001*
*Error*	*788.75*	*75*	*10.51*		
*Total*	*21,490.00*	*78*			
*Total result*	*2407.94*	*77*			

## Discussion

The results showed that the students’ knowledge about patient safety increased significantly after PBL educational approach. The results of the following studies are consistent with our study results. Meo (2013) showed that the students who were educated through the PBL method acquired significantly higher knowledge and skill compared to the students who were educated through lecture-based learning [33]. A study conducted by Yew and Goh (2016) showed that PBL is an effective teaching and learning approach, especially when evaluated for long-term knowledge retention and applications [34]. PBL is a preferential method for both the long-term retention of course content and the use of clinical skills [35]. It plays an important role in improving the knowledge horizons and learning skills and enriching the teamwork experience. Moreover, the tutor’s role as facilitators and motivators for appropriate activities is one of the main reasons for improving knowledge in PBL sessions [36]. PBL can improve nurses’ education by teaching them how to apply theory to clinical practice and develop their problem-solving skills [37]. It encourages students to be self-centered and promotes their critical thinking, leadership, and teamwork skills [38]. Dring (2019) revealed that PBL prepares students to work together and effectively communicate to provide more patient-focused care [39]. Contrary to our findings, Arpanantikul and Luecha (2010) reported that engaging in collaborative learning is considered a challenge, and the PBL method has failed to improve learning processes and knowledge acquisition. They concluded that nursing students in the PBL method discuss non-specific issues, fail to create group ideas, and obtain incomplete and superficial knowledge [40].

Our result revealed that nursing students’ attitudes toward patient safety increased significantly after the intervention. In line with our findings, Terashita et al. (2016) concluded that plain radiography practical training through PBL promoted students’ attitudes toward self-efficacy and increased their self-efficacy through self-centered learning [41]. Furthermore, Park and Choi’s (2015) study showed that PBL plays a considerable role in improving learning attitude, critical thinking disposition, and problem-solving skills in nursing students [42]. PBL improves learning by constructing an understanding of the interrelationship between basic science concepts and medical knowledge [43]. Limited studies have investigated the effect of PBL on nursing students’ knowledge and attitude toward patient safety. Liu et al. (2009) reported that the PBL approach is an effective way for nursing students to improve patient safety knowledge and enhance the integrative capacity [44]. Sahota (2020) stated that PBL promotes learners’ knowledge and skills in non-technical subjects, including patient safety, and enhances their ability to cope with the challenges they encounter in clinical environments [45].

Our findings also showed that the perception of patient safety increased significantly in nursing students after PBL education. High nursing students’ perceptions of patient safety were reported in a similar study [25]. This increased students’ perceptions of patient safety through implementing the PBL method can be explained by its significant effect on students’ learning, motivation, and experience [46]. Penjvini and Shahsawari (2013) found that students in the PBL group acquired more knowledge and had a higher level of motivation towards learning, and provided better care for patients than students in the lecture group [47]. Kim and Han (2016) showed that education programs that are implemented to strengthen critical thinking, self-efficacy, and problem-solving promote patient safety competence among clinical nurses [48]. Despite the many benefits of the PBL method, it can stress students by creating frustration, anxiety, uncertainty, and fear [49, 50]. PBL is also known as a time-consuming educational method [40, 49].

In general, Liu et al. (2019) concluded that problem-based learning is superior to the conventional teaching methods in areas such as interest in learning, teamwork spirit, problem-solving ability, analysis, knowledge attainment and application, and communicational skills [51]. Another study reported that problem-based learning enhances active learning and students’ innate motivation, which improves deep learning among students [52]. Khatiban et al. (2019) conducted a study to compare the effect of two methods of lecture-based and problem-based learning in ethics education among nursing students. They recommended problem-based learning to be used in other nursing areas since it is an effective tool for developing moral reasoning [53]. In a recent systematic review, authors have shown the effectiveness of problem-based learning in nursing education and student empowerment, so that they called for a widespread acceptance and use of this method for education in nursing schools [54].

### Study limitations

One of our study’s limitations was the participants’ mental and emotional state while completing the questionnaires and answering the questions by which the study results could be influenced. This limitation was beyond the control of the researcher. The short follow-up period was another limitation of our study. Therefore, the authors suggest other studies with a more extended follow-up period to be conducted through which the effect of the PBL educational approach on the persistence of learning over time is determined. Another limitation of the study was that nursing students were from the same nursing faculty in the control and intervention groups. We suggest students be recruited from different nursing schools in future studies. Nursing students’ pre-interventional patient safety knowledge was another weakness of this study. The authors tried to control it with a statistics test of ANCOVA.

## Conclusion

Patient safety is of great significance in various nursing education areas, including nursing education and practice. Like other healthcare team members, nursing students have the opportunity to improve the quality of patient safety. Meanwhile, nursing instructors play a vital role in improving the students’ required knowledge, attitude, and perception of patient safety. They can ensure that nursing graduates are well prepared to provide a safe environment and care for patients. Based on this study’s findings, PBL significantly impacted students’ knowledge, attitude, and perception toward patient safety compared to conventional teaching methods. Considering the PBL positive outcomes, including learning improvement, continuous and self-centered learning, concentration on understanding concepts, and innovation, it is recommended that nursing professors apply this teaching method in some courses to promote students’ clinical and cognitive capabilities to ensure safe patient care.

## Supplementary Information

**Additional file 1.**

## Data Availability

The datasets used and analyzed during the current study are available from the corresponding authors on request.

## Abbreviations

PBL: Problem-based learning
WHO: World Health Organization
SPSS: Statistical Package for the Social Sciences
ANCOVA: Analysis of covariance
CONSORT: Consolidated Standards of Reporting Trials
GPA: Grade Point Average
