# Supplementary material for: Does problem-based learning education improve knowledge, attitude, and perception toward patient safety among nursing students? A randomized controlled trial
Source: BMC Nurs. 2021 Apr 29;20:70. doi: 10.1186/s12912-021-00588-1 (PMC8086128; doi:10.1186/s12912-021-00588-1)
Supplement: Supplementary file 1 — Additional file 1. [file 12912_2021_588_MOESM1_ESM.docx]

**The PBL-based education**

Implementing the educational process in the intervention group was such that in each session, a written scenario was presented to the students about knowledge, attitude, and perception of patient safety, and students had a week to review the scenario. A problem-based learning method was implemented to investigate each scenario in the intervention group. The PBL method's steps were as follows: In the first step, the instructor asked the students to read the problem scenario and encouraged them to clarify vague concepts. In the second step, the problem was defined by the instructor. In the third step, the students had brainstorming and group discussion about the problem. In the fourth step, students listed facts and generated hypotheses based on the scenario content, and answered the questions based on the nursing process to achieve educational goals. In the fifth step, they reached a consensus on learning objectives within the group, and the instructor assured them about achieving complete, comprehensive, and appropriate goals. In the sixth step, they conducted independent and group study to gather information using the library and the internet from resources introduced. In the seventh step, the instructor presented and analyzed the solutions based on the hypotheses, goals, and questions, conducted the interdisciplinary discussion, summarized and evaluated the presented solutions. In each of the five intervention groups, eight education sessions of 45-60 minutes were conducted. Then, a total of 40 sessions was carried out using the PBL in this study. The instructor presented and reviewed the scenario delivered to the students last week at the beginning of each session. At the end of the session, the instructor presented the following week's scenario to the students.

**The routine education**

In the control group, the researcher performed routine education methods to teach the same educational content regarding patient safety. The hospital's routine method was to lecture and discuss educational content. In each control group, eight routine sessions were held during the study period.

**Patient safety training booklet**

The world health organization defines patient safety as not causing possible harm to the patient during the medical care process (WHO, 2011). Patient safety is a global health concern that affects patients in all areas of health services in all countries of the world, whether developed or developing. Research studies have shown that on average, 10% of all hospitalized patients are injured to varying degrees. At the same time, it is estimated that up to 75% of these errors are preventable. It is estimated that between 5% and 10% of health-related costs are due to unsafe clinical services that lead to patient harm. Among these, the share of failure of systems and processes is more than the role of individuals. The World Health Organization (WHO) at the Third Annual Conference on Patient Safety (October 2011) identified major adverse events in medical care in developed and developing countries as infections, unsafe surgeries, unnecessary and unsafe injections, counterfeit drugs, and communicable diseases through unsafe blood transfusion (WHO, 2011).

**Patient safety status in developing and developed countries (2011 report of the World Health Organization)**

- In developing countries, 16 billion injections are prescribed each year; 70% of them are unnecessary and could be given orally. Reuse of injection equipment has caused 260,000 HIV infections per year, resulting in 2.3 to 4.7 million new hepatitis B and C cases.
- 5-10% of patients' permanent disability and death in developing countries are due to unsafe surgeries.
- A high percentage of counterfeit drug sales in developing countries is reported (above 77%), which is less than 1% in developed countries.
- In developing countries, patients are much more likely to be injured in hospitals than in industrialized countries. The potential risk of nosocomial infections in some developing countries is about 20 times higher than in developed countries.
- In 2007, the American Nursing Association reported 48,000-98,000 deaths in US hospitals due to poor safety measures.
- Although there are no written statistics on the rate of medical errors in Iran, some studies conducted in the country have reported alarming rates of medical errors.

**Practical measures to achieve and improve patient safety:**

The UK National Health System has proposed seven steps to healthcare providers as a practical solution to achieve patient safety, and these steps are summarized below.

1. Creating a culture and creating a bed: Provide an open and fair bed to achieve patient safety.

2. Support and lead your employees: Emphasize and focus on patient safety in your organization transparently and firmly.

3. Integrate risk management operations: Create systems and processes for risk management and identify and evaluate errors.

4. Encourage and promote reporting: Assure your employees that they can easily report events locally and nationally.

5. Involve people and the community in the issue and communicate with them: Create ways to communicate openly with patients and listen to them.

6. Learn patient safety lessons and teach others: Encourage staff to radically analyze the causes to learn why and how accidents occur.

7. Implement your solutions to prevent harm: Establish and implement lessons learned by changing operations, processes, or systems.

**International Patient Safety Objectives**

The International Patient Safety Objectives were presented by the Joint International Commission (JCI) in January 2011 in the framework of valid international standards to all hospitals as follows.

1. Correct identification of patients

2. Improve effective communication

3. Improving the safety of high alert drugs (high-risk drugs)

4. Ensuring the correct position, correct working method, and the correct patient to perform surgery

5. Reduce the risk of infections acquired from health care

6. Reduce the risk of injury to the patient caused by a fall.

**Patient safety solutions**

The World Health Organization has presented nine executive strategies as solutions to achieve and improve patient safety, the titles of which are:

1. Pay attention to drugs with similar names and pronunciations to prevent drug errors

2. Pay attention to the patient's characteristics to prevent errors

3. Effective communication at the time of patient delivery

4. Perform the right procedure in the right place in the patient's body

5. Controlling the concentration of electrolyte solutions during injection

6. Ensuring the accuracy of drug therapy in the transitional stages of service delivery

7. Avoid improper connections of catheters and tubes

8. Use injection equipment only once

9. Improve hand hygiene to prevent infections related to health care

**Reduce medical errors and improve patient safety**

It is estimated that about 10% of hospitalizations result in adverse events, half of which are preventable. This is roughly equivalent to one million admissions a year of unwanted errors. Common errors include medication-incorrect medication errors, incorrect doses (e.g., adult doses to children), medication errors (e.g., intravenous cytotoxic drugs to Spinal cord), communication errors, and delayed diagnosis. Methods of reducing medical errors have largely been derived from approaches used in other sectors with more experience, especially in the aviation industry. Like the healthcare industry, the aviation industry is very complex, and any mistake in it can be fatal. But if a mistake like the health system were to occur in the aviation industry, we would see the planes crash everywhere every day. Other sectors, such as nuclear power plants, also provide examples of risk management. The model used by both industries is the "Swiss cheese" model with different layers of defense to prevent errors. Each layer may have holes, and if these holes are in the same direction, an error can occur. In medicine, there are dangerously few preservative slices of cheese, and this can lead to disaster. In industry, reporting systems are a part of the job, and the main emphasis of reporting systems is on learning from mistakes rather than blaming people. In contrast, health services have a fragmented and random reporting system in which there is no standard definition of adverse events. The culture of sin and covering it up prevails.

**Reference:**

1. Ali, Delkhoroshan; Firoozabadi, Mohammad Taghi. Clinical sovereignty. Vice Chancellor for Treatment, Birjand University of Medical Sciences and Health Services, First Edition, October 2011.

2. Abdi. Patient safety culture. Documents of lectures presented at the Conference on Patient Safety, April 27-28, 2012, Imam Hospital Hall.

3. Website of the Deputy Minister of Health of the Ministry of Health, Clinical Governance Office: http: //medcare.heapth .gov.ir.

4. Patient Safety Friendly Hospital Book Assessor's Guide, Second Edition, Ministry of Health, Treatment and Medical Education, 2012.

5. Who patient safety programme، 3 rd Annuap conference on safety ، pagos 13th and 14^th^ October 2011, Avaipabpe from: <http://www.who.int>.

6. Internationap patient safety goaps(IPSG)،pubpished 1 january 2012، Avaipabpe from:http://www.jointcommissioninternationap.org.

**Educational scenarios**

**Scenario 1**

The patient was an 18-year-old girl from one of the cities of Hamedan. She was admitted to the surgery ward with a complaint of heartburn and diarrhea. In the X-rays and colonoscopy, the evidence was in favor of ulcerative colitis, and according to the surgeon's diagnosis, the patient was a candidate for colon resection with preservation of the patient's rectum and anus. The surgical procedure is discussed with the patient (patient's father), and he agrees to the surgery. The patient does not inquire about the operation's necessity and how it is done and only wants to get rid of his annoying symptoms. He often receives information about his illness through his companions. On the day of surgery, the operating room nurse also checks the consent form in the patient medical record. During the surgery, the resident notices a mistake in the diagnosis of small bowel disease and, according to Crohn's diagnosis, tells the operating room nurse that it is not possible to preserve the patient's rectum and anus and an ileostomy should be performed for the patient, so it is necessary. Inform the senior resident that a diagnosis has been made incorrectly and ask him or her to be present in the operating room. After the senior resident's presence in the operating room, the diagnosis of Crohn's disease and the occurrence of a medical error is confirmed. The operating room's senior resident then comes out and consents to the ileostomy, the patient's most available companion (the patient's brother), and the same surgery is performed on the young girl. After recovery, the patient realizes that the ileostomy is permanent.

Investigate medical malpractice. Is it possible to prevent it? What should be done to prevent this event?

**Scenario 2**

The patient, a 55-year-old woman, was diagnosed with DKA and admitted to the ICU on 2018/09/23 at 05:55. **On 2018/09/28 at 05:51**, the doctor will order the transfer to the internal ward. After transferring to the interior (the next day), they notice bruising around the eyes and cheeks; According to the patient's companions, it happened when the patient was transferred from the ICU, but none of the ICU and internal staff observed trauma, and the patient did not say anything at the time of delivery. During the night shift in the internal ward, the patient stated that something hit me. But the nurse does not follow up on the event. The next day (transfer day) in the morning shift, a clear bruise was noticed around the patient's eye during the internal physician visit, ophthalmologist consultation is requested, and a CT scan of the axial and coronal orbit is performed. Ecchymosis, hematoma, and fracture of the left orbital wall are diagnosed. She was referred to a maxillofacial specialist after discharge.

Discuss possible reasons that caused injury to the patient. What strategies should the nurse do to prevent this kind of adverse event?

**Scenario 3**

Peyman, a 31-year-old man, was admitted with psychosis and drug abuse at 11:45. He has been started using it at the age of 23. Because the average time for psychiatric patients to respond to psych medications is about two weeks after the start. Therefore, he did not give a good answer. The patient received care in an isolated room. After admission in the morning shift, the nurse with three other staff began to provide Shift reports based on the ISBAR and Medications Administration Record (MAR) at the nursing station. The camera monitored the patient in the isolation room. The patient was sitting on the bed with his back was on the camera. The patient's hand is bloody, and after examination, they find that the patient has extracted three of his teeth using a physical restraint belt pin that is attached to an isolated bed. The belt is quickly untied, and the patient is taken to the bathroom for cleaning. The patient received haloperidol PRN every 6 hours to control and control unpredictable behaviors.

Tell me about the situation? Whose fault was here for what happened to the patient? What should the nurse do to protect the patient?

**Scenario 4**

The patient was a 6-month-old child in the pediatric ward treated with metronidazole. After starting the drug infusion, the mother referred to the nurse and stated that her child felt restless after receiving the serum. The nurse immediately went to the patient's bedside and noticed his medication error (injection of magnesium sulfate instead of metronidazole) and informed the attending doctor right away.

What was wrong here? What do you think about this medication error? Find the measures the nurse could be done to prevent medication errors.

**Scenario 5**

The patient, a 61-year-old woman, was admitted to the CCU ward. She bent to get the bedpan from the bedside floor on 2018/10/05 at 18:30 (while the side rails on both sides of the bed were elevated, without getting help or informing the nurse), which causes the patient to fall. The patient suffered a bruise on his left wrist, a graph that showed no fracture. In the area behind the patient, a hematoma with dimensions of 1 * 2 has been created. According to the doctor's instructions, a cold compress has been applied to the hematoma area after examining the patient. The patient was monitored for a brain injury for up to 24 hours. She had no signs of brain injury and was discharged after the completion of her cardiac treatment.

What was wrong in this situation? What could be done to prevent the even?

**Scenario 6**

The patient was a 32-year-old woman transferred to the operating room from the surgical ward for a cesarean section on 2018/10/31 at 10:00 AM. Before starting the cesarean section, the device sounded the alarm after connecting the catheter to the patient. The operating room staff rechecked the connections and the device. He was with the patient's skin, and after the device alarm went off spontaneously, the operation started, and no alarm was heard from the device during the surgery. However, after the procedure, the patient's leg burn and the burn site was checked by a doctor, and the burn site was washed and bandaged. After the procedure, the device was checked by medical equipment, and he was completely healthy.

Please tell me your opinion regarding this medical malpractice. What should be done to prevent it?

**Scenario 7**

Ms. Ahmadi lives in a nursing home. One night, a nurse mistakenly injected her insulin, even though she does not have diabetes. The nurse immediately realized her mistake and informed Ms. Ahmadi, her family members, and the other nurses. The care center immediately transported Ms. Ahmadi to the hospital. The nurse was praised for expressing her wrong injection. After the accident, the nurse received more training on medications to reduce the possibility of a similar error in the future.

What environmental and organizational factors made it easier for the nurse to expose medication errors and communicate with the patient and his family? What are the benefits of this communication and this honesty?

**Scenario 8**

Ms. Rezaei, a single mother, gave birth to her first child. She was a healthy baby at 37 weeks pregnant and weighed 2,700 grams. The birth was normal, and the condition of the mother and baby was stable one hour after delivery, and the nurse informed her that everything was fine. Breastfeeding began six hours after delivery. The nurse verbally told the doctor about some of the mother's breastfeeding problems and that the baby was very drowsy. The mother was released from the hospital after 36 hours. The doctor told her husband that everything was fine, and the baby had mild jaundice. He said to the family that the baby would be better in a few days. He also stated that breastfeeding improves the baby's jaundice. The doctor told Ms. Rezaei to come back to the office a week later for a check-up. After discharge, breastfeeding problems continued, and the baby's jaundice symptoms became more severe.

Ms. Rezaei was frightened and went to the emergency room when she was home for three days. The emergency room doctor asked for a bilirubin test, which was 13.5 mg/dl. He asked Ms. Rezaei to return for a visit a week later. In the following days, the baby needed to be breastfed every 1.5 hours, but Ms. Rezaei was unable to do so. Ms. Rezaei's friends, who did not have children, told her not to worry because the doctors said there was no problem.

When the child was ten days old, Ms. Rezaei brought her to the hospital. The doctor examined her, and her weight was reduced by 20%, and Bilirubin was 35 mg/dl. During the clinical examination, the child was diagnosed with bilirubin-induced encephalopathy. The hospital's advisory board tried to understand how this preventable condition had progressed.

What happened in this case? Where were the highlights? What measures could be taken to prevent this from happening?
